# *TEAD4* is a novel independent predictor of prognosis in LGG patients with *IDH* mutation

**DOI:** 10.1515/biol-2021-0039

**Published:** 2021-04-08

**Authors:** Hai-Yan Yuan, Ya-Juan Lv, Yi Chen, Dan Li, Xi Li, Jian Qu, Han Yan

**Affiliations:** Department of Pharmacy, The Second Xiangya Hospital, Central South University, Changsha 410011, People’s Republic of China; Department of Clinical Pharmacology, Xiangya Hospital, Central South University, Changsha 410008, People’s Republic of China; Institute of Clinical Pharmacology, Central South University, Changsha 410078, People’s Republic of China

**Keywords:** low-grade glioma, copy number variation, *TEAD4*, prognosis, gene expression

## Abstract

TEA domain family members (TEADs) play important roles in tumor progression. Till now, the genomic status of *TEADs* in patients with glioma has not been well investigated. To confirm whether the genomic status of *TEADs* could affect the prognosis of patients with glioma, the copy number variation (CNV), mutation and expression data of glioma cohorts in The Cancer Genome Atlas, Gene Expression Omnibus and Chinese Glioma Genome Atlas were comprehensively analyzed. Results showed that *TEAD* CNV frequency in lower grade gliomas (LGGs) was higher than in glioblastoma multiforme (GBM). Multivariate cox regression analysis showed that *TEAD4* CNV increase was significantly associated with overall survival (OS) and disease-free survival (DFS) in LGGs (OS *p* = 0.022, HR = 1.444, 95% CI: 1.054–1.978; DFS *p* = 0.005, HR = 1.485, 95% CI: 1.124–1.962), while not in GBM. Patients with *TEAD4* CNV increase showed higher expression level of *TEAD4* gene. In LGG patients with *IDH* mutation, those with higher TEAD4 expression levels had shorter OS and DFS. Integrating *TEAD4* CNV increase, *IDH* mutations, *TP53* mutation, *ATRX* mutation and 1p19q co-deletion would separate patients with LGG into four groups with significant differences in prognosis. These study results suggested that *TEAD4* variations were independent predictive biomarkers for the prognosis in patients with LGG with *IDH* mutation.

## 1 Introduction

Malignant glioma is a primary brain tumor with extremely high mortality in adults [1,2,3]. Glioblastoma multiforme (GBM; World Health Organization grade IV) is notorious for resistance to therapy and has mean survival of less than 15 months [4,5]. Diffuse low-grade and intermediate-grade gliomas together make up the LGGs (lower grade gliomas), including World Health Organization grades II and III [6]. Majority of the patients with LGGs are sensitive to therapy and experience extended survival depending on the molecular subtype, such as *IDH* mutant and 1p19q co-deletion [7,8]. While the current curative effect and the prognosis of LGG varies greatly (survival time ranging from 1 to 15 years) due to individual differences, a certain number of patients could not gain satisfactory prognosis [9,10].

Central nervous system tumor diagnosis has entered the molecular era since 2016, which is defined by both histology and molecular features. The molecular parameters contain *IDH* mutation, *ATRX* loss, *TP53* mutation, etc., and these more precisely defined the entities that are expected to improve therapeutic efficacy, clinical trials and more specific classification [11]. Also, more followed studies focus on searching molecular markers for objective diagnosis and accurate clinical outcomes. Xiao et al. constructed a CD44-related four-gene signature that would well predict the prognosis and effectively distinguish high- and low-risk patients with LGGs [12]. Nevertheless, the signatures associated with stratification of prognosis in patients with LGG remain finite, and identifying novel biomarkers is still essential for improving the diagnostic accuracy and therapeutic efficacy.

Transcription enhancer factors are the most important DNA-binding partner in Hippo and Wnt pathways, and four proteins have been identified, which are named *TEAD*1–4. When YAP/TAZ, the key molecules in the downstream of Hippo/Wnt pathway, translocate into the nucleus, *TEADs* directly interact with them and mediate the main transcriptional output of the Hippo/Wnt pathway and then drive cancer cell survival, proliferation, invasive migration and metastasis [13,14]. Hippo and Wnt/β-catenin pathways have been reported as pivotal signaling pathways, regulating cell proliferation and differentiation, immune response and subsequently facilitating tumorigenesis [15,16,17,18,19,20,21]. Meanwhile, YAP/TAZ-*TEAD* activation may also confer resistance to chemotherapy, radiotherapy or immunotherapy [22,23,24,25]. Therefore, *TEADs* might be a crucial target for glioma therapy.

Till now, the genomic status of *TEADs* in patients with glioma was not well investigated. Xu et al. discovered that the overexpression of *TEAD4* correlated with poor prognosis of glioma, but they ignored the impact of recognized factors such as *IDH* mutation and 1p19q co-deletion on the prognosis of glioma and had not considered the difference in outcomes between LGGs and GBM [26]. Simultaneously, Wang et al. conducted a comprehensive study to explore the molecular characterization of the Hippo-signaling pathway in 33 cancers [27]. They found that *TEAD2–4* were significantly correlated with LGG survival, but the detailed information of the relationship between *TEADs* and glioma patients’ outcome was not clearly elaborated. In this study, we are going to take the public data for comprehensively analyzing the relationship between *TEADs* and glioma prognosis and expecting to provide a novel strategy for individualized medicine for glioma in the future.

## 2 Methods and materials

### 2.1 Study samples

**Figure 1:**
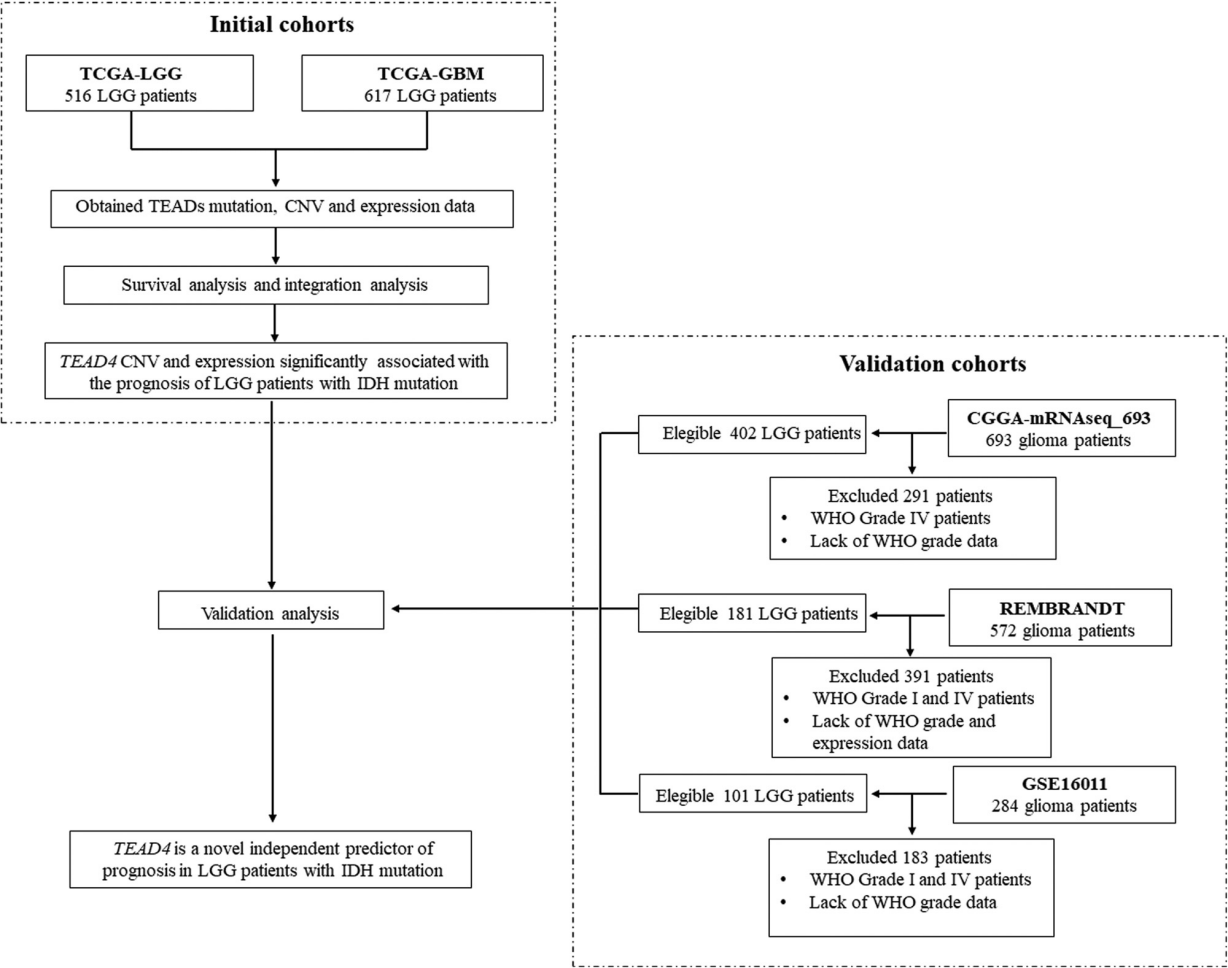
Flow diagram of the study patients.

The glioma data set used in this study included TCGA-LGG, TCGA-GBM, CGGA-mRNAseq_693, Rembrandt and GSE16011. Data of somatic mutation, copy number variation (CNV), gene expression and clinical phenotypic in TCGA-LGG and TCGA-GBM data sets were obtained from cbioportal (http://www.cbioportal.org) and the GDC database (https://portal.gdc.cancer.gov/). Data of isocitrate dehydrogenase (*IDH*) mutations, 1p19q co-deletion status, gene expression and clinical phenotypes in CGGA-mRNAseq_693 were derived from the CGGA database (http://www.cgga.org.cn/). Both of the Rembrandt and GSE16011 data set used Affymetrix gene chip technology to detect gene expression, and the data were downloaded from the GEO database (https://www.ncbi.nlm.nih.gov/geo/). The LGG samples of this study refer to samples of WHO II and WHO III levels [6]. Figure 1 displays the flow diagram of the study patients.

### 2.2 Statistical analysis

In this study, Kaplan–Meier (KM) analysis was used to perform univariate survival analysis to determine the effects of *TEAD* mutations, CNV and gene expression on the overall survival (OS) or disease-free survival (DFS) in patients with LGG. Multivariate survival analysis was performed through cox regression analysis. To select the covariates included in the multivariate survival analysis, backward stepwise cox regression analysis was conducted. The candidate covariates contained *IDH* mutation, *TP53* mutation, *ATRX* mutation, 1p19q co-deletion, tumor grade and age. Mann–Whitney analysis was utilized to test the relationship between *TEAD* CNVs and their expression. In CNV analysis, the patients with high-level threshold values of 2 or −2 (calculated by GISTIC 2.0) were considered to have a copy number change. For gene expression data, we took the median value as the grouping basis for the high expression group and the low expression group. All the analyses in this study were performed by SPSS20 and R 3.6.1. GraphPad Prism 6.0 software was utilized for drawing the survival curves and histograms. R package maftools were used to conduct mutation interaction analysis.

### 2.3 Coexpression and gene ontology (GO) enrichment analysis

We conducted coexpression analysis in carriers of *IDH* mutation and wild-type patients in both TCGA-LGG and CGGA-mRNAseq_693 LGG data sets. And then filtered the genes that significantly correlated with *TEAD4* based on Pearson correlation method (Pearson |*r*| > 0.4; Bonferroni corrected *p* < 0.05). The screened genes were finally used to perform GO analysis by David 6.8 (https://david.ncifcrf.gov/tools.jsp).

### 2.4 Immune infiltration analysis

To test the relationship between *TEAD4* expression and immune infiltration, TIMER database (http://timer.cistrome.org/) was utilized. Infiltration scores of six types of immune cells in patients with LGG were calculated. Then the correlation of *TEAD4* expression and these scores were tested.

## 3 Results

### 3.1 Basic characteristics of the study data sets

The TCGA-LGG data set included 516 patients with glioma, and the data of RNA sequencing, CNV and whole-genome somatic mutations were available for all 506 patients. The TCGA-GBM data set included 617 glioblastoma samples, among them 401 patients had somatic mutation information, 599 patients obtained whole-genome CNV data and 521 patients got whole-genome expression information by Affymetrix U133 microarray. OS and DFS data of all the patients in TCGA were available. The CGGA-mRNAseq_693 data set contained 693 patients, including 402 patients with LGG. The RNA sequencing data, *IDH* mutation data, 1p19q co-deletion status and OS information were available in this data set. Rembrandt data set contained 572 patients, and 181 were LGG. GSE16011 contained 284 patients including 109 with LGG. Both of Rembrandt data set and GSE16011 used Affymetrix microarray to obtain the whole-genome expression data of patients with glioma. OS was recorded in Rembrandt and GSE16011 data sets. Table 1 summarizes the basic characteristics of each study sample.

**Table 1:** Basic characteristics of the study data sets

Characteristics	TCGA-LGG	TCGA-GBM	CGGA-mRNAseq_693	Rembrandt	GSE16011
Sample size	516	617	693	572	284
Expression detection platform	Illumina	Affymetrix	Illumina RNA	Affymetrix	Affymetrix
	RNA seq	Microarray	RNA seq	Microarray	Microarray
CNV data	Available	Available	NA	NA	NA
*IDH* mut data	Available	Available	Available	NA	NA
1p19q codel data	Available	Available	Available	NA	NA
*TP53* and *ATRX* mutation data	Available	Available	NA	NA	NA
Prognostic phenotype	OS, DFS	OS, DFS	OS	OS	OS

### 3.2 Mutation and CNV frequency analysis

TCGA data set showed that the incidence of somatic mutations in *TEADs* in gliomas was extremely low, as only 0.3% (3/812) of the patients was carrying the mutations. Meanwhile *TEAD* CNV occurred in LGG with a frequency of 11.7% (60/513), and in GBM with a frequency of 2.9% (17/577). Interestingly, the frequency of *TEAD* CNVs in LGG was significantly higher than that in GBM (*p* = 1.83 × 10^−8^). *TEAD4* was the main CNV of all *TEAD* CNVs, accounting for 48.3% (29/60) in LGG and 58.8% (10/17) in GBM. The *TEAD4* CNV occurrence in LGG was also significantly higher than that of GBM (*p* = 5.06 × 10^−4^). The CNV increase in *TEAD4* was the main form of *TEAD4* CNV in both LGG and GBM (28/29 in LGG and 9/10 in GBM).

### 3.3 Survival analysis of patients with *TEAD* CNV

**Figure 2:**
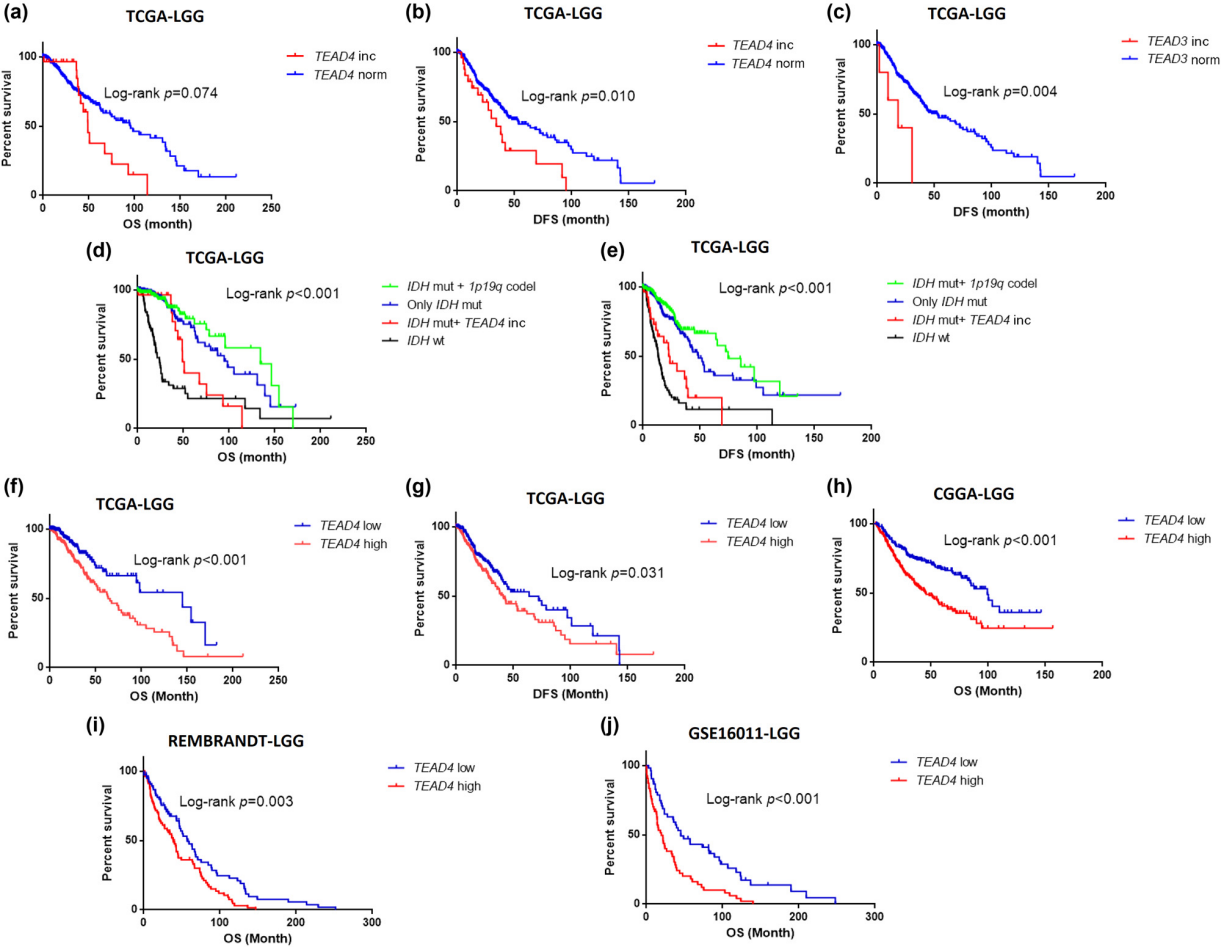
Survival curve of *TEAD3* and *TEAD4* in patients with LGG. (a)–(e) Survival curve for CNVs (*TEAD*3 and *TEAD*4) in TCGA-LGG cohort. (f)–(j) Survival curve for *TEAD4* expression. Blue lines represent gene normal copy number (norm) or *TEAD4* low expression, and red lines represent gene copy number increase (inc) or *TEAD4* high expression. OS means overall survival and DFS means disease-free survival.

**Figure A1:**
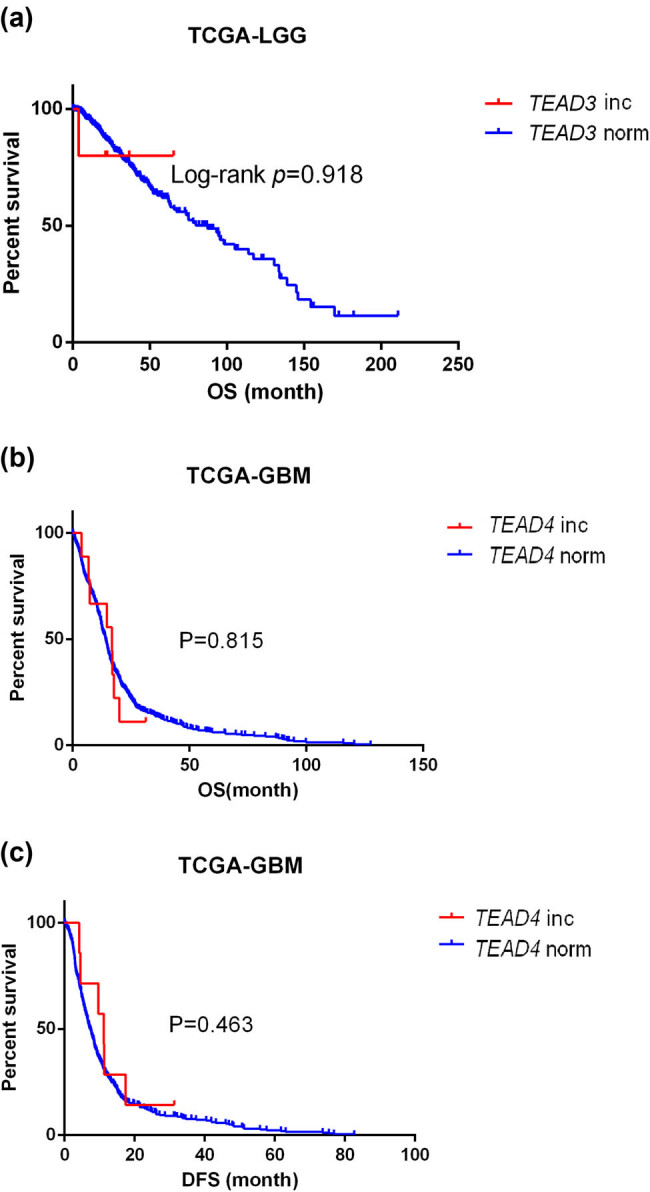
Survival Curve of *TEAD3* and *TEAD4* CNV in patients with glioma. (a) Overall survival curve for *TEAD3* CNV in TCGA-LGG cohort. (b) Overall survival curve for *TEAD4* CNV in TCGA-GBM cohort. (c) Disease-free survival curve for *TEAD4* CNV in TCGA-GBM cohort. Blue lines represent gene normal copy number (norm), while red lines represent gene copy number increase (inc). OS means overall survival, and DFS means disease-free survival.

Survival analysis showed that *TEAD4* CNV was strongly related to OS and DFS in LGG. The median OS of patients with *TEAD4* CNV was obviously shorter than that of patients without *TEAD4* CNV (*p* = 0.074, HR = 1.288, 95% CI: 0.973–1.703) (Figure 2a). And meanwhile, the median DFS for patients without *TEAD4* CNV was significantly longer than the patients carrying *TEAD4* CNV (*p* = 0.010, HR = 1.382, 95% CI: 1.074–1.778) (Figure 2b). In GBM, *TEAD4* CNV was uncorrelated with OS and DFS (OS *p* = 0.815, DFS *p* = 0.463) (Figure A1b and c). Similarly, *TEAD3* CNV was also significantly associated with DFS in LGG (*p* = 0.004, HR = 1.961, 95% CI: 1.190–3.230) (Figure 2c) but not OS (*p* = 0.918) (Figure A1a). Only one *TEAD3* CNV carrier was found in patients with GBM. In contrast, *TEAD1* and *TEAD2* showed no association with glioma prognosis (Table 2).

**Table 2:** Association analysis results between *TEAD* CNVs and prognosis in glioma

Gene	LGG					GBM				
	INC	NOR	DEC	OS *p*	DFS *p*	INC	NOR	DEC	OS *p*	DFS *p*
*TEAD1*	2	497	14	0.165	0.988	0	575	1	NA	NA
*TEAD2*	1	496	16	0.791	0.132	3	573	1	0.857	0.882
*TEAD3*	5	508	0	0.918	**0.004**	2	576	0	NA	NA
*TEAD4*	28	484	1	**0.074**	**0.010**	9	567	1	0.815	0.463

Note: *p* value was calculated by log-rank method, the *p* values less than 0.1 were in bold. If the sample size of a group was less than 3, the group was deleted. INC represents CNV increase; NOR represents normal; DEC represents CNV decrease.

Backward stepwise cox regression analysis selected 1p19q co-deletion, *TP53* mutation, tumor grade and age as the covariates for OS; and *IDH* mutation, 1p19q co-deletion and age as the covariates for DFS. After adjusting for covariates, *TEAD4* CNV increase significantly remains associated with OS (*p* = 0.022, HR = 1.444, 95% CI: 1.054–1.978) and DFS (*p* = 0.005, HR = 1.485, 95% CI: 1.124–1.962) in patients with TCGA-LGG (Table 3). These results indicated that *TEAD4* CNV increase was an independent predictor of LGG prognosis.

**Table 3:** Univariate and multivariate analysis results of *TEAD4* CNV in TCGA-LGG cohort

	OS				DFS			
	Univariate		Multivariate		Univariate		Multivariate	
	*p*	OR (95% CI)	*p*	OR (95% CI)	*p*	OR (95% CI)	*p*	OR (95% CI)
*TEAD4* CNV	0.077	1.288 (0.973–1.703)	0.022	1.444 (1.054–1.978)	0.012	1.382 (1.074–1.778)	0.005	1.485 (1.124–1.962)
Age	6.39 × 10^−15^	1.058 (1.043–1.074)	1.02 × 10^−11^	1.056 (1.040–1.073)	4.35 × 10^−5^	1.026 (1.013–1.039)	0.001	1.023 (1.010–1.037)
Grade	1.58 × 10^−9^	3.340 (2.258–4.940)	6.99 × 10^−5^	2.264 (1.513–3.386)	0.003	1.608 (1.174–2.202)		
*IDH* mutation	2.22 × 10^−22^	0.156 (0.108–0.227)			2.54 × 10^−21^	0.178 (0.124–0.254)	5.19 × 10^−11^	0.245 (0.161–0.373)
*TP53* mutation	0.039	0.690 (0.485–0.982)	2.42 × 10^−7^	0.294 (0.185–0.468)	0.791	1.042 (0.767–1.417)		
*ATRX* mutation	0.070	0.714 (0.497–1.028)			0.802	1.040 (0.764–1.416)		
1p19q co-deletion	1.21 × 10^−4^	0.411 (0.261–0.646)	3.41 × 10^−11^	0.166 (0.098–0.282)	4.64 × 10^−6^	0.408 (0.278–0.599)	0.008	0.557 (0.362–0.856)

Integrated analysis of *TEAD4* CNV increase, *IDH* mutations, *TP53* mutation, *ATRX* mutation and 1p19q co-deletion showed that the patients with LGG would divide into four groups with different prognosis. Group 1 (*n* = 167) included the patients with both *IDH* mutation and 1p19q co-deletion, and they had the best outcomes. Group 2 (*n* = 203) included the patients with *IDH* mutation but without either *TEAD4* CNV increase or 1p19q co-deletion, and their outcomes were only second to group 1. Most of the group 2 patients carried *TP53* mutation and/or *ATRX* mutation, and only two patients had neither *TP53* mutation nor *ATRX* mutation. Group 3 (*n* = 27) included patients with both *IDH* mutation and *TEAD4* CNV increase but not 1p19q co-deletion, whose prognosis was significantly worse than the patients with LGG having *IDH* mutations while without *TEAD4* CNV increase (OS *p* = 0.016, HR = 1.465, 95% CI: 1.065–2.016; DFS *p* = 0.014, HR = 1.405, 95% CI: 1.067–1.850). All of the group 3 patients carried at least one of these two mutations (*TP53* mutation and *ATRX* mutation). Group 4 (*n* = 96) included the patients with *IDH* wild-type LGG, and they had significantly worse outcomes than group 3 patients (Figure 2d–e).

### 3.4 Co-occurrence analysis of *TEAD4* CNV, *IDH* mutation, *TP53* mutation, *ATRX* mutation and 1p19q co-deletion

**Figure 3:**
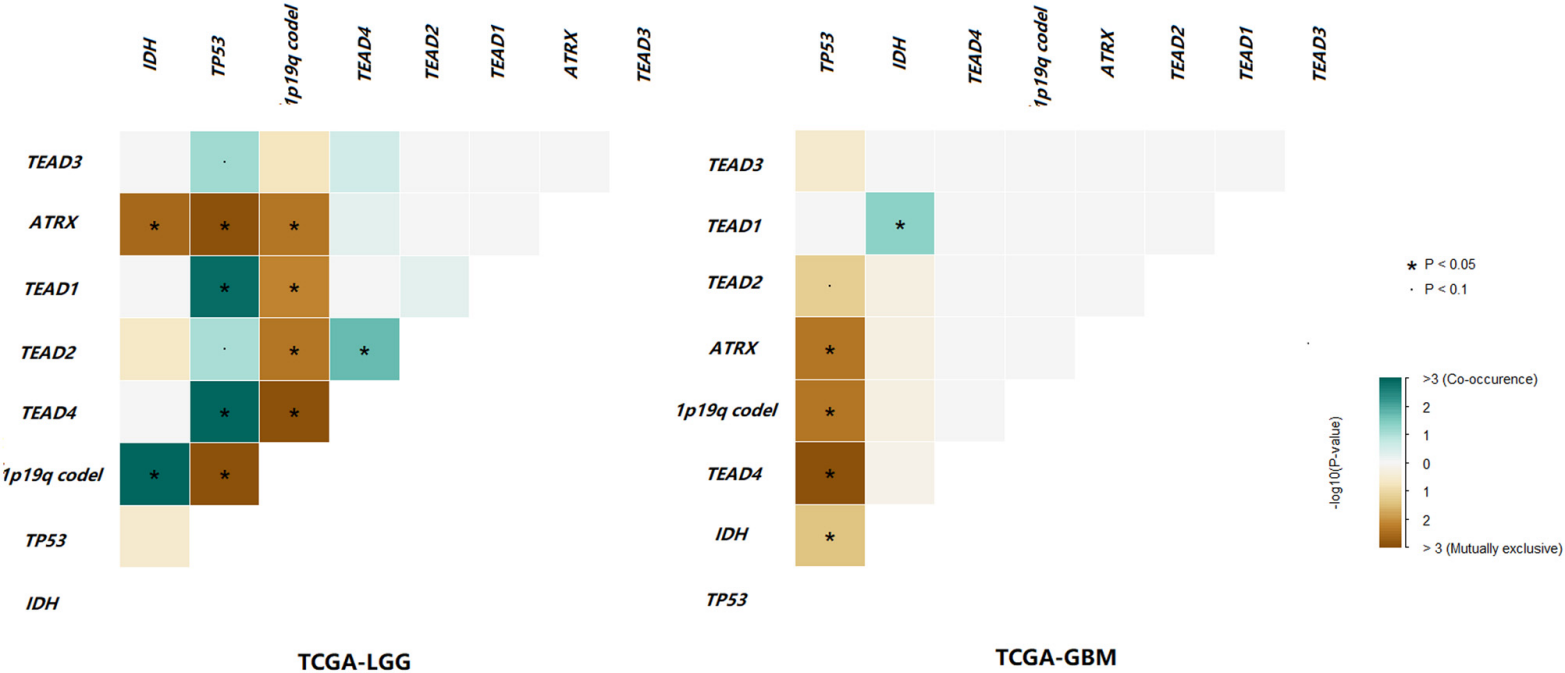
Interactions of *TEAD* CNVs, isocitrate dehydrogenase (*IDH*) mutation, *TP53* mutation, *ATRX* mutation and 1p19q co-deletion in TCGA-LGG and TCGA-GBM cohorts. Green represents co-occurrence and brown represents exclusive.

To find the reasons for the diverse clinical effects of *TEAD4* CNV increase on LGG and GBM, we conducted a series of explorations, including mutation co-occurrence analysis. Results showed that *TEAD4* CNV increase and *TP53* mutations were significantly mutually exclusive in GBM, while it was opposite in LGG (Figure 3). In addition, *TEAD4* CNV increase and 1p19q co-deletion were significantly mutually exclusive in LGG, while this phenomenon did not occur in GBM. Moreover, 28/29 *TEAD4* CNV increase carriers simultaneously had *IDH* mutation in LGG, while only 1/9 *TEAD4* CNV increase carriers had *IDH* mutation in GBM.

### 3.5 *TEAD4* CNV was associated with *TEAD4* expression

**Figure 4:**
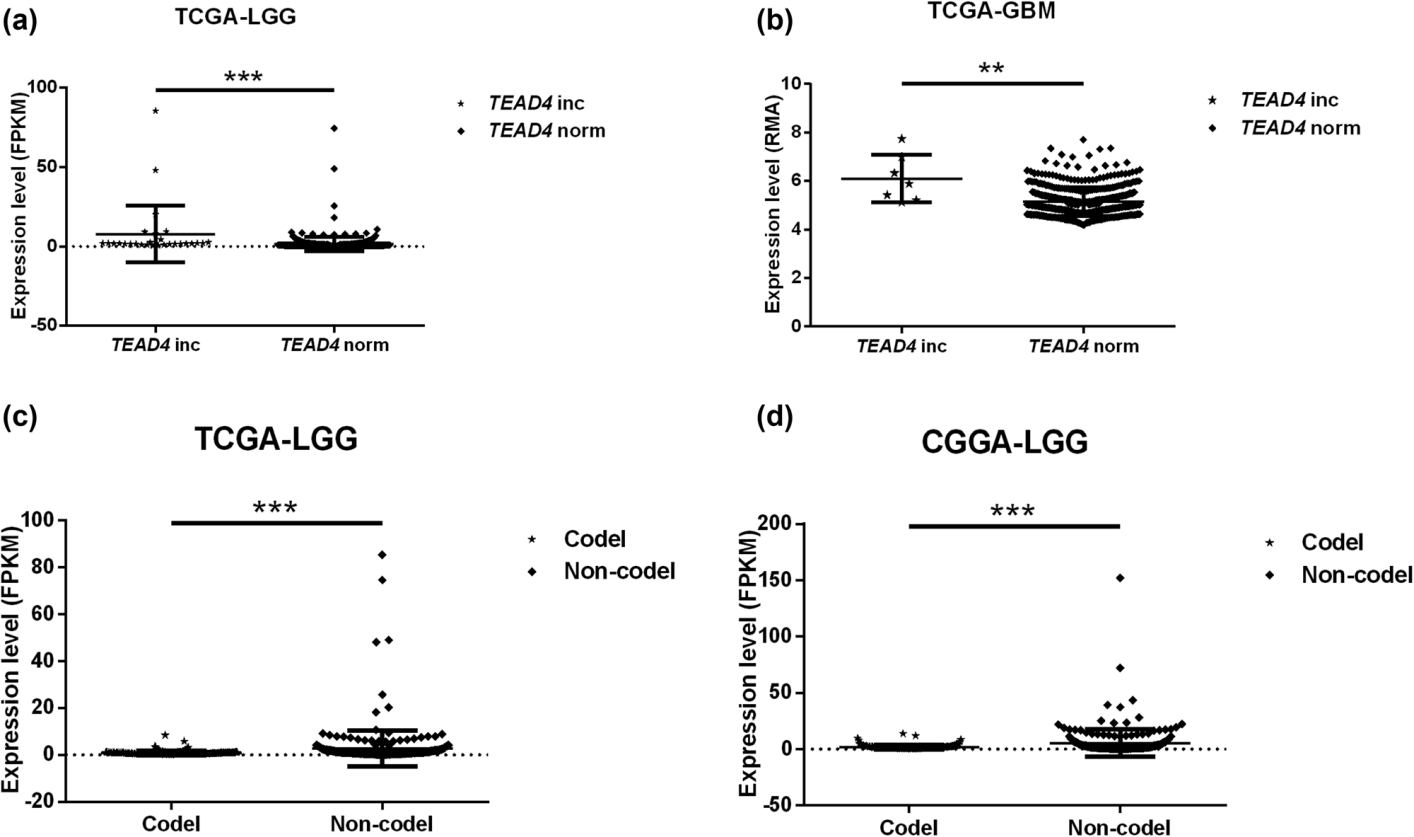
Distribution of *TEAD4* expression according to its CNV and 1p19q co-deletion. (a) and (c) TCGA-LGG cohort. (b) TCGA-GBM cohort. (d) CGGA-mRNAseq_693 LGG cohort. Norm represents *TEAD4* normal copy number, inc represents *TEAD4* copy number increase. Codel represents 1p19q co-deletion and noncodel represents lack of 1p19q co-deletion. FPKM means the expression data were normalized by FPKM method, while RMA means the expression data were normalized by RMA method.

Differential expression analysis showed that carriers with *TEAD4* CNV increase had a higher *TEAD4* expression level than the normal *TEAD4* copy number carriers (LGG *p* = 8.27 × 10^−7^; GBM *p* = 0.004) (Figure 4a and b; FPKM means the expression data were normalized by FPKM method; and RMA means the expression data were normalized by RMA method [28,29]). On the contrary, the *TEAD3* CNV was not significantly associated with the expression of *TEAD3* (LGG *p* = 0.077; GBM *p* is unavailable).

**Figure A2:**
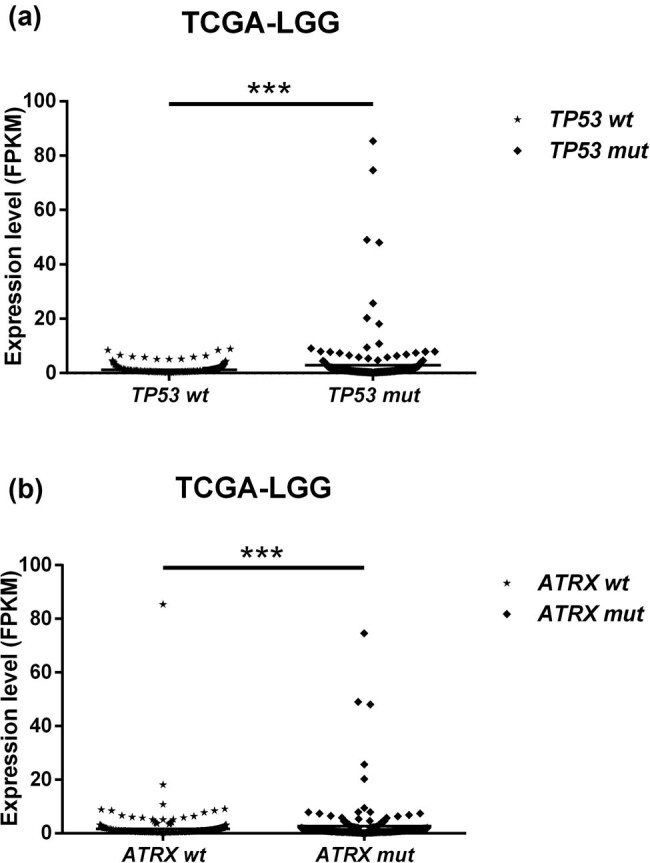
Distribution of *TEAD4* expression according to *TP53* mutation and *ATRX* mutation. mut represents mutation, wt represents wild type, FPKM means the expression data were normalized by FPKM method.

The relationship between *TEAD4* expression and *IDH* mutation, *TP53* mutation, *ATRX* mutation or 1p19q co-deletion was also tested. We found that the expression of *TEAD4* was significantly downregulated in both TCGA-LGG and CGGA-mRNAseq_693 LGG cohorts when 1p19q co-deletion occurred (Figure 4c and d). The *TEAD4* expression level was irrelevant to *IDH* mutation in patients with both of TCGA-LGG and CGGA-mRNAseq_693 LGG. In addition, the expression of *TEAD4* was also found to be significantly upregulated in *TP53* mutation or *ATRX* mutation carriers in TCGA-LGG (Figure A2). None of *TP53* mutation and *ATRX* mutation information was provided in CGGA-mRNAseq_693 data sets.

### 3.6 *TEAD4* expression was an independent prognosis predictor for patients with LGG carrying *IDH* mutation

Survival analysis showed that *TEAD4* expression was significantly associated with OS and DFS in TCGA-LGG samples (Figure 2f and g). The median survival time of the high *TEAD4* expression group was dramatically shorter than that of the low *TEAD4* expression group (63.500 months vs 144.940 months; *p* = 7.71 × 10^−5^, HR = 2.113, 95% CI: 1.446–3.088). Similarly, the median DFS time was markedly shorter in patients with high *TEAD4* expression than the low *TEAD4* expression group (41.060 months vs 72.170 months; *p* = 0.022, HR = 1.431, 95% CI: 1.050–1.949). Similar results were found in CGGA mRNAseq_693-LGG, Rembrandt-LGG and GSE16011-LGG (Figure 2h and j). The expression of *TEAD4* was irrelevant to OS and DFS in patients with TCGA-GBM (OS *p* = 0.815, DFS *p* = 0.890).

To confirm whether *TEAD4* expression level was an independent factor for the prognosis of LGG, multivariate cox regression analysis was conducted in TCGA-LGG and CGGA mRNAseq_693 LGG data sets. After adjusting for covariates, the expression level of *TEAD4* was only significantly associated with OS in CGGA mRNAseq_693 LGG population (Table 4).

**Table 4:** Survival analysis of *TEAD4* expression in *IDH* mutant and *IDH* wild-type patients

Data set	*IDH* mutation status	*N*	Adjusted HR (95% CI)	Adjusted *p*
OS				
TCGA-LGG	ALL	505	1.325 (0.867–2.024)	0.193*
	***IDH mut.***	**409**	**2.226 (1.260–3.935)**	0.006**
	*IDH wt.*	96	0.869 (0.473–1.596)	0.651***
CGGA-mRNAseq_693	ALL	402	1.666 (1.201–2.311)	**0.002^#^**
LGG	***IDH mut.***	**306**	**1.805 (1.216–2.679)**	**0.003** ^**##**^
	*IDH wt.*	96	1.218 (0.730–2.031)	0.450^###^
DFS				
TCGA-LGG	ALL	505	1.066 (0.766–1.3482)	0.706^&^
	*IDH mut.*	409	1.128 (0.759–1.677)	0.550^&&^
	*IDH wt.*	96	0.824 (0.455–1.492)	0.523^&&&^

Note: *Adjusted by age, 1p19q co-deletion, *TP53* mutation and tumor grade; **adjusted by age, 1p19q co-deletion and tumor grade; ***adjusted by age and tumor grade; ^#^adjusted by 1p19q co-deletion, *IDH* mutation and tumor grade; ^##^adjusted by 1p19q co-deletion and tumor grade; ^###^adjusted by tumor grade; ^&^adjusted by age, 1p19q co-deletion and *TP53* mutation; ^&&^adjusted by 1p19q co-deletion; ^&&&^adjusted by age and *ATRX* mutation. All covariates were selected by backward stepwise cox regression analysis. The *p* values reaching to the edge of a significant level (<0.1) were in bold.

Based on stratified survival analysis of *TEAD4* expression according to *IDH* mutation status, we found that *TEAD4* expression significantly affected the OS in patients with LGG carrying *IDH* mutants after adjusting for covariates. On the other hand, in *IDH* wild-type LGG patients, *TEAD4* expression had no correlation with the outcomes. These results were validated in CGGA mRNAseq_693 population (Table 4). The results suggested that *TEAD4* expression might be an independent predictor of prognosis for patients with LGG carrying *IDH* mutation, and the *TEAD4* gene function might have a synergistic effect with the *IDH* mutation.

### 3.7 Coexpression and GO enrichment analysis

A total of 91 genes were found significantly coexpressing with *TEAD4* in patients with both TCGA-LGG and mRNAseq_693 with *IDH* mutations. All of these genes were positively correlated with *TEAD4*. In carriers of *IDH* wild-type, 420 genes were found significantly coexpressing with *TEAD4*, and all genes were positively correlated with *TEAD4*. Among these genes, 41 coexpressed with *TEAD4* only in *IDH* mutation carriers, while 370 coexpressed with *TEAD4* only in *IDH* wild-type patients (Tables S1 and S2).

GO enrichment analysis suggested that the top 20 GO terms enriched by 41 genes were mainly related to immune and membrane, such as T-cell receptor-signaling pathway, MHC class II protein complex, plasma membrane and so on (Table S3). However, the top 20 GO terms enriched by 370 genes were mainly correlated with binding, such as protein binding, integrin binding, actin filament binding and so on (Table S4).

### 3.8 Immune infiltration analysis

**Figure 5:**
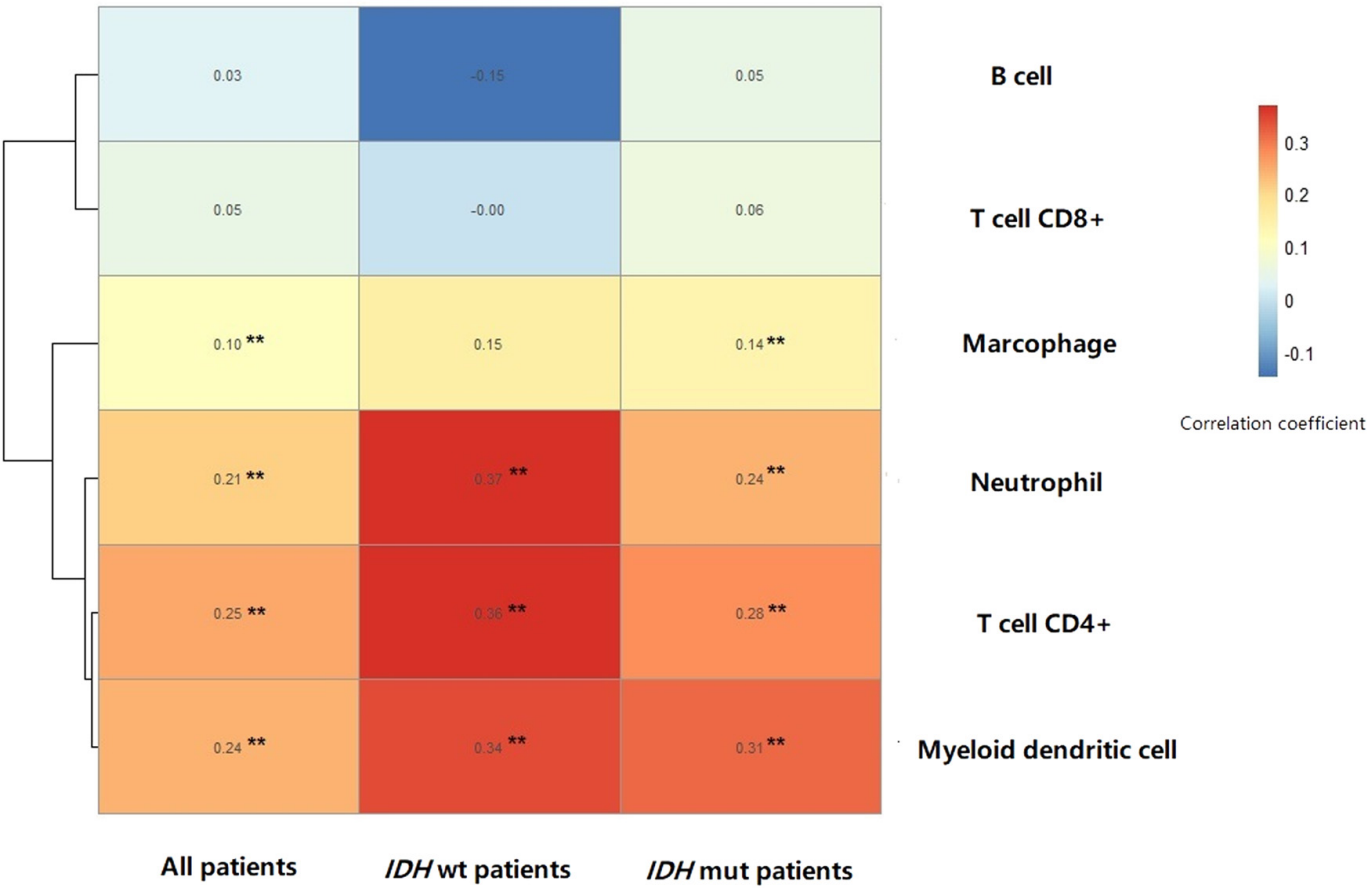
Heat map of the correlation coefficient of immune infiltration scores and *TEAD4* expression.

The expression level of *TEAD4* was significantly positively correlated with immune infiltration scores of myeloid dendritic cell, T-cell CD4^+^, neutrophil and macrophage in patients with TCGA-LGG. The same results were found in patients with TCGA-LGG carrying *IDH* mutation. In patients with TCGA-LGG carrying wild-type *IDH* mutation, except for macrophage, the immune infiltration scores of myeloid dendritic cell, T-cell CD4^+^ and neutrophil significantly positively correlated with *TEAD4* expression (Figure 5). No significant difference was observed between *IDH* mutation and *IDH* wild-type patients.

## 4 Discussion

In this study, we found that *TEAD* CNVs had a higher incidence in LGG than in GBM. Additionally, *TEAD4* CNV which strongly regulated *TEAD4* expression was significantly associated with the outcomes of patients with LGG. Interestingly, we’ve discovered that both *TEAD* CNVs and *TEAD* expressions were taking effect only in patients with LGG, but not in patients with GBM. Meanwhile, we also found that *TEAD4* CNV increase and *IDH* mutations might be mutually exclusive in GBM; while in LGG, these two mutations occur synergistically. Moreover, integrating *TEAD4* CNV, *TP53* mutation, *ATRX* mutation, *IDH* mutations and 1p19q co-deletion would separate patients with LGG into four groups with different prognosis, which might provide a new biomarker for developing new therapeutic regimens to improve the outcomes in patients with LGG carrying *IDH* mutations but suffering poor prognosis.

In our study, we discovered that *TEAD4* CNV increase might lead to poor prognosis in patients with LGG carrying *IDH* mutations, but not in patients with LGG not carrying *IDH* mutation or the patients with GBM. This compelling phenomenon might most probably be due to the *IDH* mutation that could promote the synthesis of 2-hydroxyglutarate and then lead to hypermethylation phenotype in cells which would regulate lots of gene expression levels in various pathways [30,31,32]. So there might be significant differences in the activity of many signaling pathways including Hippo and Wnt between *IDH* mutation and wild-type individuals.

Meanwhile, we conducted *TEAD4* coexpression analysis in *IDH* mutant and *IDH* wild-type patients, and pathway analysis was performed for the relating genes. The results suggested enormous difference in *TEAD4* coexpression genes between *IDH* mutation and wild-type patients. In *IDH* mutation carriers, the *TEAD4* correlating genes mainly enriched in immune response–related pathways; while in *IDH* wild-type patients, the genes concentrated in other biological pathways. The reported *IDH* mutant could alter the tumor immunological microenvironment in LGGs, and the immune system gene signature could predict the prognosis of glioma [32,33]. Several studies found that YAP/TAZ expression could regulate the cross-talk between immune cells and tumor cells in the tumor microenvironment through binding to *TEADs* and then suppressing the T-cell viability and triggering tumor immune evasion [34,35,36].

Based on these, the expression of *TEAD4* would take on different roles in glioma prognosis depending on whether the patient is carrying *IDH* variation. As we know, *IDH* mutation carriers in LGG tend to have longer survival than other types of gliomas [37]. Nevertheless, some of these patients are still suffering poor prognosis, while the reasons were unknown yet. The influence of CNV on clinical outcome is ubiquitous in various malignant tumors including gliomas [38]. The level of total genomic CNV inversely correlated with both PFS and OS in *IDH*-mutant LGG (grades II and III) [39,40,41]. Our results suggested that *TEAD4* CNV increases and the high expression level would dramatically aggravate outcomes in patients with LGG carrying *IDH* mutations, which might partly explain why these patients are experiencing poorer prognosis.

In the meantime, our study discovered that 1p19q co-deletion would downregulate *TEAD4* expression in glioma. However, *TEAD4* CNV and expression level could affect the prognosis in LGG independent of 1p19q. *TEAD4* is located on chromosome 12, suggesting that its decreased expression is not caused by the deletion of chromosome 1p or 19q directly. The specific reason for the correlation of 1p19q co-deletion with *TEAD4* expression is not clear at present, and further functional studies are needed to determine it.

Our research results were somewhat different from those reported by Xu et al. as mentioned in the Introduction section [26]. Our study found that CNV and overexpression of *TEAD4* only affected the prognosis in patients with LGG carrying *IDH* mutation. The reasons for this difference might be that Xu et al. did not distinguish the discrepancy of prognosis and genes expression between LGG and GBM, and they had not considered the effects of *IDH* mutation, *TP53* mutation, *ATRX* mutation and 1p19q on the prognosis of glioma. In addition, Xu et al. found that the expression of *TEAD4* was negatively correlated with *IDH1* mutation; while our study found that *TEAD4* expression level was irrelevant to *IDH* mutation in patients with LGG [26]. This might because they did not take into account that the frequency of *IDH* mutation was extremely low in GBM and the relatively higher level of *TEAD4* expression in GBM. So it appeared that *IDH1* mutation was significantly negatively related with *TEAD4* expression.

Nevertheless, our study has several limitations. First of all, in addition to *TEAD4*, the frequency of other *TEAD* CNV was very low, and thus we could not confirm the influence of *TEAD1–3* in prognosis of patients with LGG. Second, the frequency of *IDH* mutation in GBM was very low, hence we could not explore whether the interaction between *TEAD4* CNVs and *IDH* mutation in GBM is the same as in LGG. Finally, the results in this study were acquired from clinical data analysis; therefore, further experiments were required for validation.

## 5 Conclusion

This study discovered that CNV and gene expression status of *TEAD4* were closely related to the prognosis of patients with LGG carrying *IDH* mutation. Incorporating *TEAD4* CNVs might better stratify the patients with LGG, which would provide new biomarkers for establishing new molecular classification systems for further precision medicine in glioma.
